# HCV Core Antigen Diagnostic Performance for Acute/Recent HCV Infection in People With HIV-1: A Systematic Review and Meta-Analysis

**DOI:** 10.1093/ofid/ofaf576

**Published:** 2025-10-17

**Authors:** Tsung-Yu Tsai, Guan-Jhou Chen, Hsin-Yun Sun, Chien-Ching Hung

**Affiliations:** Department of Internal Medicine, National Taiwan University Hospital and National Taiwan University College of Medicine, Taipei, Taiwan; Department of Internal Medicine, National Taiwan University Hospital and National Taiwan University College of Medicine, Taipei, Taiwan; Min-Sheng General Hospital, Taoyuan, Taiwan; Department of Internal Medicine, National Taiwan University Hospital and National Taiwan University College of Medicine, Taipei, Taiwan; Department of Internal Medicine, National Taiwan University Hospital and National Taiwan University College of Medicine, Taipei, Taiwan; Department of Internal Medicine, National Taiwan University Hospital Yunlin Branch, Yunlin, Taiwan; Department of Tropical Medicine and Parasitology, National Taiwan University College of Medicine, Taipei, Taiwan

**Keywords:** negative likelihood ratio, negative predictive value, positive likelihood ratio, positivity predictive value, reinfection, sexually transmitted infection

## Abstract

**Background:**

Hepatitis C virus (HCV) core antigen (HCVcAg) has been validated to identify HCV infection, but its efficacy in diagnosing acute HCV infection among people with HIV-1 (PWH) warrants further investigation. This systematic review and meta-analysis aimed to evaluate the diagnostic performance of HCVcAg for acute or recent HCV infection among PWH.

**Methods:**

We conducted a literature search to identify studies assessing the diagnostic performance of HCVcAg from January 2020 to May 2024. Acute or recent HCV infection was defined as the presence of negative anti-HCV antibody but positive nucleic acid amplification tests (NAATs), or anti-HCV antibody seroconversion within 1 year. Studies were included if they evaluated HCVcAg diagnostic accuracy using NAATs as the gold standard and provided sufficient data for sensitivity and specificity assessment. Studies lacking a clear definition of acute or recent infection were excluded.

**Results:**

Four out of 229 articles met our inclusion criteria, with 1015 participants providing 1796 tests. The sensitivity of HCVcAg to diagnose acute or recent HCV infection ranged from 87.1% to 100% and specificity from 95.0% to 100%. The meta-analysis yielded a pooled sensitivity of 0.92 (95% CI, 0.78–0.98) and specificity of 0.99 (95% CI, 0.97–1.00). Based on the global incidence (8.46 per 1000 person-years) of HCV viremia among PWH, the positive and negative predictive values of HCVcAg were 0.44 (95% CI, 0.18–1.00) and 1.00 (95% CI, 0.99–1.00), respectively.

**Conclusions:**

HCVcAg has good diagnostic performance in identifying PWH with acute or recent HCV infection, supporting its integration into HCV screening protocols for PWH.

Hepatitis C virus (HCV) infection is a major cause of chronic hepatitis, which triggers persistent inflammation and subsequent liver damage. The estimated global prevalence of viremic HCV infection is around 0.7%, representing 56.8 million active cases [1]. These cases serve as viral reservoirs and contribute to onward viral transmission, presenting a substantial obstacle to disease elimination. The advent of direct-acting antivirals (DAAs) against HCV, with high sustained virological response (SVR) rates, has made the elimination of hepatitis C feasible. Consequently, the World Health Organization has established a goal to end the epidemic of viral hepatitis by 2030 [2]. Given the exceptional efficacy and effectiveness of DAAs, strategic focus has shifted toward test-and-treat approaches targeting high-risk populations [3, 4], including men who have sex with men (MSM), people with HIV-1 (PWH) [5–8], people who inject drugs (PWID) [9, 10], and commercial sex workers [11, 12].

Among high-risk populations, PWH are particularly vulnerable, with a greater disease burden in Eastern Europe and Sub-Saharan Africa. The global prevalence of HCV infection among PWH is estimated at up to 6.3%, whereas the global incidence in this population is estimated to be 8.46 per 1000 person-years [13]. Individually, PWH face accelerated progression of HCV-related complications, evidenced by higher incidences of cirrhosis, hepatocellular carcinoma, and decompensated liver failure [14–16]. HCV reinfections, despite effective DAA treatments, remain a global challenge [17–21]. Moreover, undiagnosed acute HCV infection among PWH continues to be a critical gap in HCV elimination as these individuals may unknowingly transmit HCV to sexual contacts via high-risk sexual behaviors, undermining the health of PWH and the ambitious goal to end the HCV epidemic [17]. Thus, early identification of PWH with acute HCV infection and prompt initiation of DAA treatment are essential components of comprehensive HCV elimination programs.

Traditionally, the diagnosis of HCV infection relies on anti-HCV antibody and nucleic acid amplification tests (NAATs). In clinical practice, anti-HCV testing is used to screen symptomatic individuals with suspected HCV exposure, followed by confirmatory NAATs. However, the use of anti-HCV in the timely diagnosis of acute hepatitis C is limited by the longer seroconversion window periods in PWH [18, 19] and its inability to detect HCV reinfection. Although NAATs remain the gold standard for detecting acute HCV infection in PWH, their accessibility is often restricted in resource-limited settings or low- and middle-income countries due to financial considerations [20]. Consequently, a cost-effective diagnostic approach is needed in practice.

The highly conserved protein HCV core antigen (HCVcAg) has been developed as a detection target for HCV infection, offering a shorter assay time and reduced costs when repeat testing is required [21]. This commercially available HCVcAg test is a potentially suitable diagnostic tool for such settings. Existing systematic reviews and meta-analyses have demonstrated the excellent diagnostic performance of HCVcAg in active HCV infection [22–24]. However, the utility of HCVcAg specifically for acute HCV infection among PWH remains incompletely characterized. Thus, we aimed to evaluate the diagnostic performance of HCVcAg in the context of acute or recent HCV infection among PWH.

## METHODS

We conducted a systematic review investigating published studies that evaluated the diagnostic performance of the HCVcAg assay for acute or recent HCV infection among PWH. Data from the selected studies were gathered and analyzed through a meta-analysis to synthesize diagnostic performance, including sensitivity, specificity, positive likelihood ratio (PLR), and negative likelihood ratio (NLR). Our methodology adhered to the PRISMA-DTA Statement guidelines [25], following a predetermined protocol for literature search, study selection, data extraction, risk of bias and quality evaluation, and statistical analysis. The study was registered with the International Platform of Registered Systematic Review and Meta-analysis Protocols (INPLASY; registration number INPLASY202530106).

### Search Strategy

We examined PubMed, EMBASE, Scopus, and Web of Science (WoS) to identify English-language studies that assessed the diagnostic performance of HCVcAg from January 2000 to May 2024. We utilized search terms encompassing HCV, core antigen, HIV, sensitivity, and specificity. All methodological details, including the population, intervention, comparison, outcomes, and study design framework, inclusion and exclusion criteria, and search strategy, are provided in the Supplementary Data. We also conducted a citation search of existing systematic reviews and meta-analyses to identify additional eligible studies [22–24].

Two authors (T.Y.T. and G.J.C.) independently evaluated article eligibility and conducted a full-text review. The senior author (H.Y.S.) resolved any discrepancy in assessment.

### Study Selection

Our inclusion criteria comprised (1) PWH either undergoing acute HCV infection testing or with available anti-HCV data to establish acute HCV infection; (2) case–control, cross-sectional, cohort, or randomized controlled studies; (3) using ARCHITECT HCV Ag assay (Abbott, Germany) as the diagnostic tool and NAATs as the reference standard; and (4) available numbers of true positive (TP), false positive (FP), true negative (TN), and false negative (FN), preferably listed as a table. Studies were excluded if they (1) lacked any data on sensitivity, specificity, TP, FP, TN, or FN; (2) provided insufficient data on timing of HCV infection; (3) aimed to evaluate treatment response to DAAs; (4) utilized antibody or antibody–antigen combination tests; or (5) consisted only of conference abstracts with unpublished data.

PWH with acute HCV infection were defined as individuals with the presence of a documented seroconversion of anti-HCV within 1 year or negative anti-HCV but positive HCV viremia.

### Data Extraction and Quality Assessment

Two authors (T.Y.T. and G.J.C.) independently evaluated the included studies and extracted relevant data, with a senior author (H.Y.S.) resolving any disagreement. Quality was assessed using the Quality Assessment of Diagnostic Accuracy Studies 2 (QUADAS-2) tool [26] (Supplementary Data).

### Statistical Analysis

The statistical analysis was performed using STATA, version 18 (StataCorp, College Station, TX, USA) to calculate the pooled sensitivity, specificity, PLR, and NLR with 95% CIs for HCVcAg, using NAATs as the reference standard. Results were visualized through forest plots and summary receiver operating characteristic (sROC) curves. To simulate real-world clinical applications of HCVcAg, we incorporated global and local epidemiological data of HCV infection among PWH [13] to calculate their positive predictive value (PPV) and negative predictive value (NPV), presenting post-test probability through Fagan plots. The analysis employed the STATA command *midas,* based on a bivariate random-effects meta-analysis model [27, 28], with heterogeneity assessed by Higgins & Thompson's *I*² statistic, where values of 25%, 50%, and 75% indicate low, moderate, and high heterogeneity, respectively [29].

## RESULTS

### Search Results

**Figure 1.**
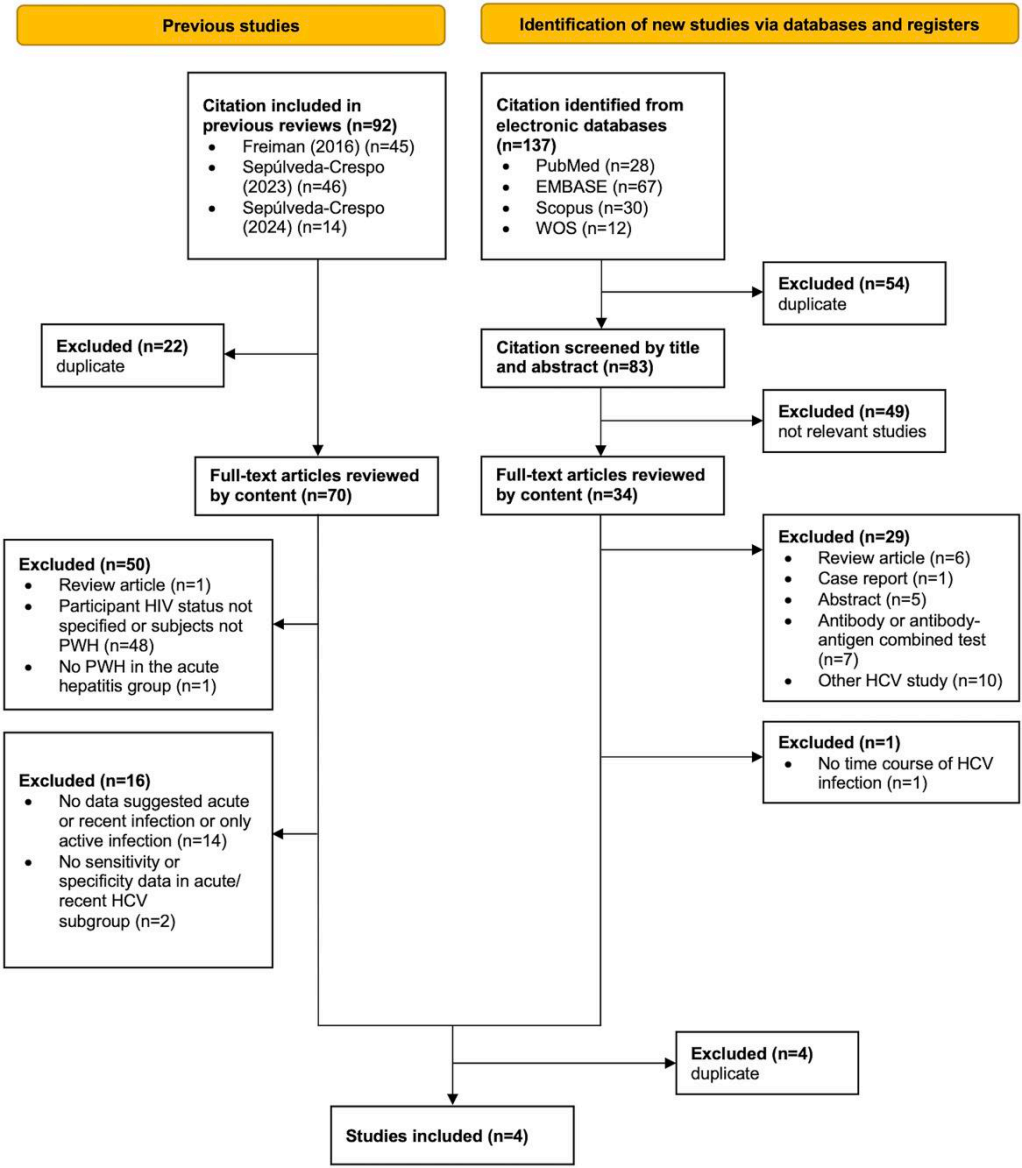
The study selection process. Abbreviations: HCV, hepatitis C virus; PWH, people with HIV; WOS, Web of Science.

The initial search identified 229 potentially eligible studies, comprising 137 studies retrieved from 4 electronic databases and 92 studies from 3 previously conducted meta-analyses [22–24]. Figure 1 illustrates the study selection process. Of the 137 database-sourced studies, 54 were excluded because of duplication and 49 were excluded after title and abstract screening. Four studies were eligible after full-text review. Of the 92 studies from existing meta-analyses [22–24], 22 were excluded because of duplication, and 4 were qualified after comprehensive review. These 4 studies consisted of 1015 participants who contributed 1796 tests to calculate the pooled sensitivity, specificity, PLR, and NLR.

### Study Characteristics

All 4 studies were conducted in developed countries. One employed a prospective design while 3 utilized retrospective methodologies. Table 1 details the demographic characteristics of each study. All used ARCHITECT HCV Ag assay as the diagnostic tool, and 3 employed a cutoff value of 3 femtomoles per liter (fmol/L) while 1 used 10 fmol/L. For the reference standard of NAATs, 3 studies employed either Cobas AmpliPrep HCV Test, version 2.0 (Roche, Pleasanton, CA, USA), or Cobas 6800 (Roche, Pleasanton, CA, USA). One study did not mention the detection limit of its NAAT, and the remaining 3 utilized NAATs with a detection limit of 15 international unit per milliliter (IU/mL).

**Table 1.** Demographic Characteristics of Patients in the Included Studies Assessing the Diagnostic Performance of the HCVcAg Assay

Authors [Reference]	Year	Location	Study Design	Case Number	Population	Definition of Acute/Recent HCV Infection	Method of HCVcAg	Reference Standard	Totals	TP	FP	FN	TN	Sensitivity, %	Specificity, %
Cresswell et al. [30]	2015	United Kingdom	Prospective (an HIV outpatient clinic)	111 PWH	PWH who had elevated ALT	Testing of previously stored samples	ARCHITECT HCV Ag (positive: >10 fmol/L)	Real-time qRT-PCR (Abbott; LOD not mentioned)	111	15	2	0	94	100	97.9
Van-hommerig et al. [31]	2015	The Netherlands	Retrospective (anonymous survey in an Amsterdam STI clinic)	93 PWH	PWH in routine HCV screening in an STI clinic	Samples in widow period (anti-HCV-negative) from PWH	ARCHITECT HCV Ag (positive: >3 fmol/L)	Cobas CAP/CTM V2 (Roche Diagnostics; LOD 15 IU/mL)	65	5	3	0	57	100	95.0
Hullegie et al. [32]	2017	The Netherlands	Retrospective (testing samples of a prospective study)	67 PWH	PWH who had elevated ALT	Samples in widow period (anti-HCV-negative) and testing of previously stored samples from PWH	ARCHITECT HCV Ag (positive: >3 fmol/L)	Cobas CAP/CTM V2 (Roche Diagnostics; LOD 15 IU/mL)	67	39	0	5	23	88.6	100
Sun et al. [33]	2022	Taiwan	Retrospective (testing samples of a prospective study)	744 PWH	High-risk PWH with STIs within past 6 mo with elevated aminotransferases with a history having achieved spontaneous HCV clearance with a history of sustained virological response by antivirals	Serial testing of HCV RNA and reviewing the anti-HCV serostatus in the EMR	ARCHITECT HCV Ag (positive: >3 fmol/L)	Cobas CAP/CTM V2 before and Cobas 6800 after 2020/09 (Roche Diagnostics; LOD 15 IU/mL)	1553	54	8	8	1483	87.1	99.5

Abbreviation: EMR, electrical medical record; FN, false negative; FP, false positive; HCVcAg, hepatitis C virus core antigen; LOD, limit of detection; PWH, people with HIV; STI, sexually transmitted infection; TN, true negative; TP, true positive.

The definition of acute HCV infection varied across these studies. A prospective study at an HIV outpatient clinic enrolled 111 PWH with abnormal liver function test (LFT) results in London, United Kingdom, and acute HCV infection of 15 PWH was determined through testing of previously stored samples [30]. A retrospective study in a sexually transmitted infection (STI) clinic in Amsterdam, the Netherlands, included 93 PWH, and 31 tested positive for HCV viremia, with 5 categorized as having acute HCV infection given negative anti-HCV results [31]. The Dutch Acute HCV in HIV Study (DAHHS) included PWH with abnormal LFT results, and 44 acute HCV infections were detected in 67 PWH by negative anti-HCV and retrospective testing of stored samples [32]. Another study in Taiwan retrospectively tested samples of high-risk individuals from a prospective study of 3-stage pooled-plasma HCV RNA testing [33, 34]. It included 830 high-risk participants (744 PWH and 86 HIV-negative) who provided 1639 samples, with 62 cases of acute HCV infection identified by serial HCV NAAT testing and review of anti-HCV serostatus in electronic medical records. We contacted the authors for detailed data and excluded the 86 HIV-negative individuals and their samples. A total of 744 PWH provided 1553 samples for final analysis, with 62 samples of acute or recent HCV infection.

### Quality Assessment

The QUADAS-2 quality assessment results are shown in Supplementary Figure 1. All 4 studies demonstrated low risk of bias in the index test domain. In the patient selection domain, 1 study had an unclear risk of bias because of 86 HIV-negative individuals providing an unknown number of samples that were analyzed simultaneously [33]; therefore, we contacted the authors for detailed data. In the reference standard domain, 1 study had an unclear risk of bias given unspecified NAAT cutoff values for HCV viremia detection [30]. In the flow and timing domain, 1 study exhibited high risk of bias due to inconsistent follow-up durations (ranging from 3 to 6 months) and changes in the reference standard during its study period [33].

### Diagnostic Performance

**Figure 2.**
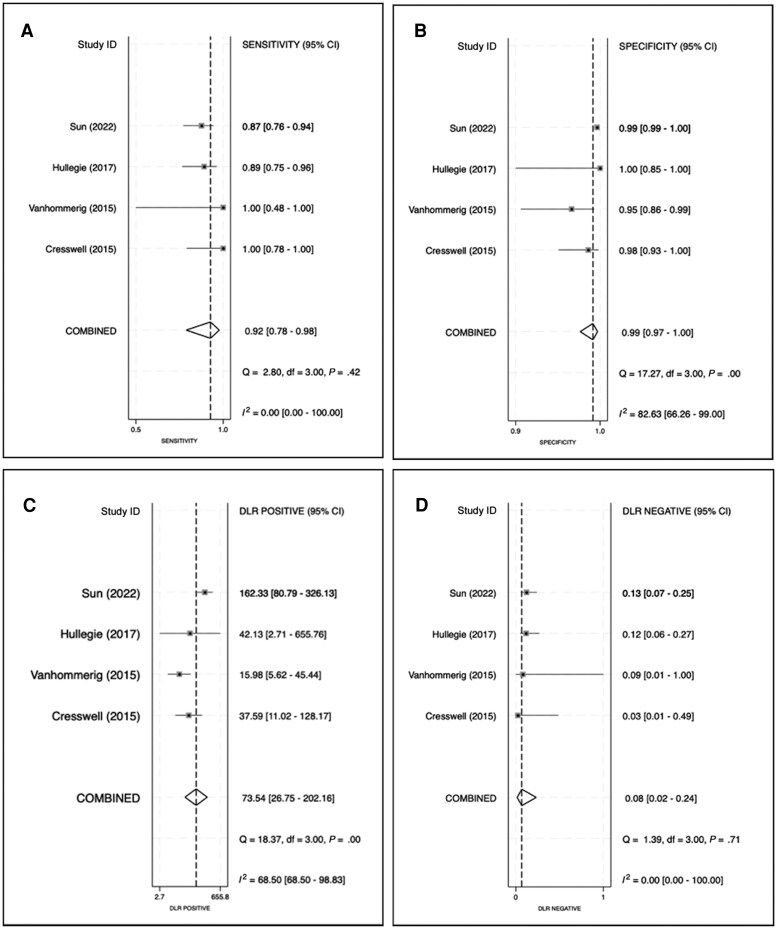
The results of forest plots of the meta-analysis. The pooled (*A*) sensitivity, (*B*) specificity, (*C*) PLR, and (*D*) NLR of the HCVcAg assay from the included studies. Abbreviations: HCVcAg, hepatitis C virus core antigen; NLR, negative likelihood ratio; PLR, positive likelihood ratio.

**Figure 3.**
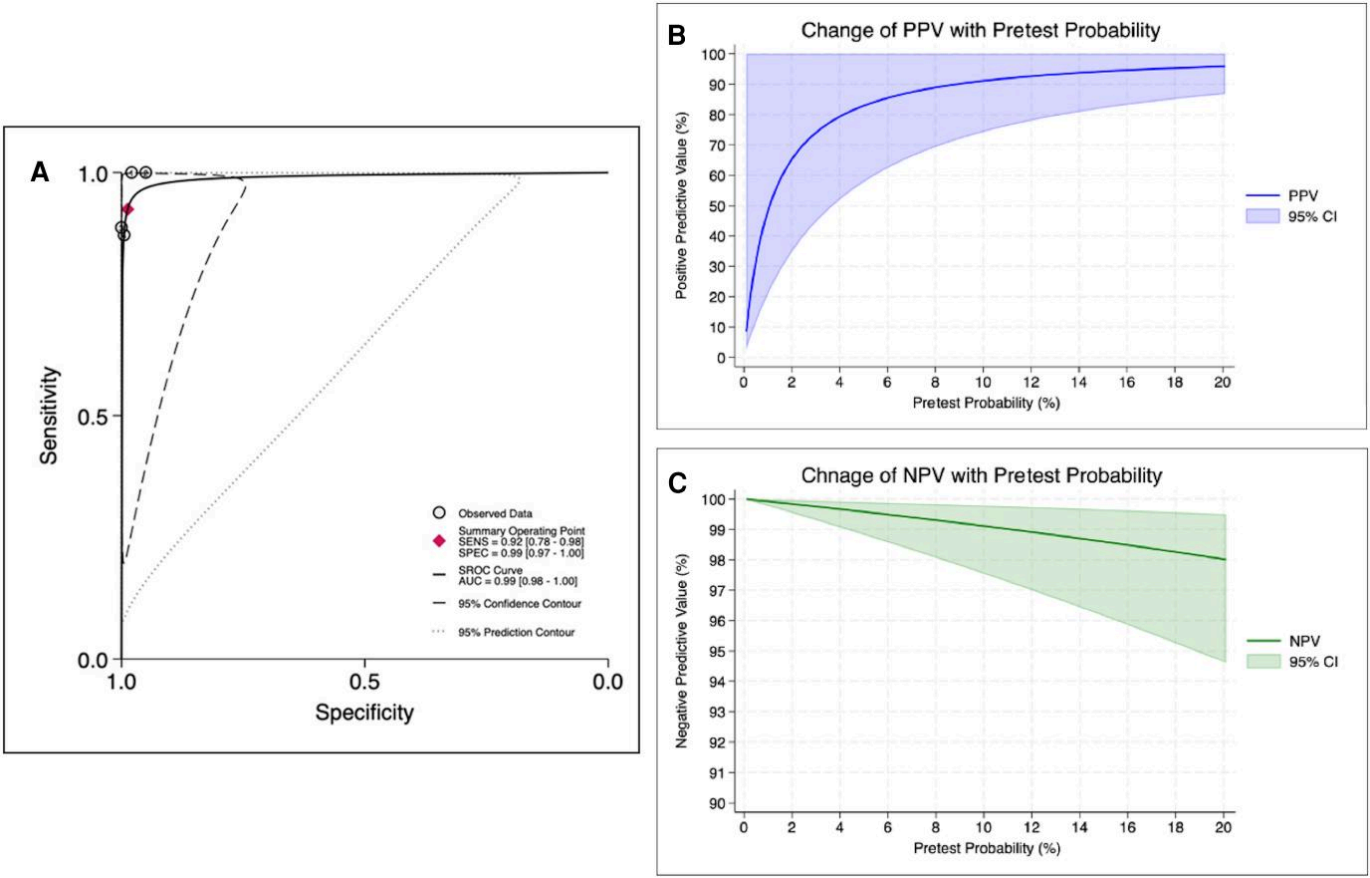
(*A*) sROC curve of the HCVcAg assay and a simulation of (*B*) sensitivity and (*C*) specificity with the alteration of pretest probability when <20%. Abbreviations: AUC, area under the curve; HCVcAg, hepatitis C virus core antigen; sROC, summary receiver operating characteristic.

The sensitivity of the HCVcAg test in the diagnosis of acute or recent HCV infection across the 4 included studies ranged from 87.1% to 100%, while the specificity ranged from 95.0% to 100%. In bivariate random-effects meta-analysis via the STATA command “*midas,*” the pooled sensitivity was 0.92 (95% CI, 0.78–0.98) (Figure 2*A*) and the pooled specificity was 0.99 (95% CI, 0.97–1.00) (Figure 2*B*). The pooled PLR and NLR were 73.54 (95% CI, 26.75–202.16) and 0.08 (95% CI, 0.02–0.24), respectively (Figure 2*C* and *D*). The sROC curve is shown in Figure 3*A*. Given the global incidence rate of HCV infection of 8.46 per 1000 person-years among MSM with HIV [13], the PPV and NPV of the HCVcAg test were 0.44 (95% CI, 0.18–1.00) and 1.00 (95% CI, 0.99–1.00), respectively. As the majority of the included participants were from the Taiwan study, the PPV and NPV were 0.40 (95% CI, 0.16–1.00) and 1.00 (95% CI, 0.99–1.00), respectively, based on the local annual incidence of 0.73% of HCV infection among MSM with HIV in Taiwan in 2022 [35]. With the notably high incidence rate of HCV reinfection of 49.30 per 1000 person-years in this population, the PPV and NPV were 0.83 (95% CI, 0.57–1.00) and 1.00 (95% CI, 0.99–1.00), respectively [35]. Supplementary Figure 2 presents the corresponding Fagan plots, whereas Figure 3*B* and *C* illustrates the changes of PPV and NPV with alteration of pretest probability.

### Heterogeneity

The overall heterogeneity was low to moderate, with an *I*^2^ value of 38 (95% CI, 0–100). The *I*^2^ values for sensitivity and specificity were 0 (95% CI, 0–100) and 82.63 (95% CI, 66.26–99.00), respectively. For PLR and NLR, the *I*^2^ values were 68.50 (95% CI, 68.50–98.83) and 0.00 (95% CI, 0–100), respectively. The heterogeneity was high for specificity and moderate to high for PLR.

## DISCUSSION

In our systematic review and meta-analysis with a total of 1796 HCVcAg tests from 1015 participants who were PWH across 4 studies, we demonstrate that the HCVcAg assay exhibits robust diagnostic performance in the diagnosis of acute or recent HCV infection, with a pooled sensitivity of 0.92 and specificity of 0.99. The pooled PLR and NLR were 73.54 and 0.08, respectively.

The clinical application of ARCHITECT HCV Ag assay to diagnose active HCV infection has been well established in previous meta-analyses [22, 24], supporting its implementation in clinical practice [36]. The European Association for the Study of the Liver (EASL) has recommended the use of HCVcAg to detect chronic infection or reinfections [37], although this recommendation has not been adopted by other guidelines [38, 39]. While similar diagnostic efficacy has been observed among PWH [23], few studies have investigated its utilization in the context of acute HCV infection, given the complexities of case definition and patient enrollment.

For instance, the Centers for Disease Control and Prevention (CDC) 2020 case definition for acute hepatitis C consists of both clinical and laboratory criteria [40]. The clinical criteria include jaundice, total bilirubin ≥3.0 mg/dL, or serum alanine aminotransferase (ALT) >200 IU/L without a more likely diagnosis, and the laboratory criteria require either positive NAATs for HCV viremia or presence of HCVcAg. However, acute HCV infection is mostly asymptomatic among PWH [41], making the diagnosis of acute infection largely dependent on routine screening. Meanwhile, acute HCV infection is typically defined as the presence of HCV viremia or HCVcAg without anti-HCV (samples in window period), unless participants undergo frequent monitoring to document anti-HCV seroconversion. In the included studies, 2 used samples in the window period (negative anti-HCV) from PWH [31, 32], 1 employed prospectively collected samples for NAAT testing [33], and all 4 were conducted in well-structured institutions with regular laboratory examinations, highlighting the challenges of infrastructure in identifying those with acute HCV infection. Nevertheless, our meta-analysis confirms that the HCVcAg assay demonstrated acceptable diagnostic performance for acute HCV infection.

Symptom-driven testing or routine regular screening is essential for PWH [39, 42], as timely diagnosis of acute HCV infection remains challenging in HCV elimination efforts. Diagnostic delays during acute infection may impede the identification of people with high HCV RNA loads, hindering the implementation of “treatment-as-prevention” strategies to control HCV transmission. Before the incorporation of HCVcAg into clinical application, several limitations remain, including the timing of testing, that it’s not a point-of-care modality, the balance of cost-effectiveness, and its performance in detecting HCV reinfection. Nevertheless, strategies such as incorporating HCVcAg into screening programs and reflex HCVcAg testing in high–anti-HCV seroprevalence areas have shown promising outcomes [43, 44].

In the included studies, symptom-driven testing was not utilized. Two studies selected participants with abnormal LFT results [30, 32], 1 conducted regular HCVcAg testing [31], and 1 employed complex inclusion criteria followed by periodical testing [33]. The optimal testing strategy should balance local epidemiology, national health policy priorities, available on-site personnel, and accessible resources. Both NAATs and HCVcAg meet the laboratory criteria of acute HCV infection, but HCVcAg may offer financial advantages [45, 46]. Although HCVcAg offers cost advantages and greater accessibility in resource-limited settings compared with NAATs, the ideal testing scheme and cost-effectiveness of this approach specifically for identifying acute HCV infection in PWH require further investigation. Our study provides preliminary evidence to support such research initiatives.

A test's PPV is directly influenced by the pretest probability. For the HCVcAg test in PWH, the low PPV of 0.44 (with a wide CI) mainly reflects the low annual incidence of initial HCV infection, which is only 8.46 per 1000 person-years globally. However, as shown in Figure 3*B* and *C*, the PPV increases with a higher pretest probability. This is particularly relevant for HCV reinfection, where epidemiological studies indicate a high incidence rate of 49.30 per 1000 person-years in PWH [35, 47–50], with an estimated PPV of 0.83. Nevertheless, the real-world effectiveness of HCVcAg in detecting HCV reinfection remains unclear. It has been observed that PWH who had spontaneous viral clearance of initial HCV infection demonstrated higher rates of spontaneous clearance during reinfection episodes [48], suggesting potential variability of viral dynamics and immune responses among individuals. Three studies addressed the cases of reinfection, with a total of 3 out of 15 (20.0%) [30], 9 out of 44 (20.4%) [32], and 28 out of 62 (45.2%) [33], but accuracy assessments for reinfections were not conducted. The remaining study used different HCVcAg cutoff values (10.00 fmol/L and 3.00 fmol/L, respectively, for reactive and at least weakly reactive samples, but the presence of reinfection was not specified) to evaluate HCVcAg’s performance, but observed no change in diagnostic accuracy [31]. While the impact of reinfection incidence on the diagnostic performance of HCVcAg testing requires further investigation, the overall diagnostic performance still provided compelling evidence for identifying acute HCV infection and warrants investigations into the potential limitations associated with reinfections.

This study has several limitations. First, we exclusively included studies focusing on using HCVcAg for diagnosis of acute or recent HCV infection, excluding studies involving monitoring of treatment responses. We aimed to explore the clinical utility of the HCVcAg assay, and studies involving treatment response evaluation exhibit considerable heterogeneity in study designs and regimens, warranting separate analysis. Second, the small number of included articles precluded sensitivity analysis. However, we have comprehensively reviewed previous meta-analyses and their included articles to minimize the risk of omission. Third, the limited number and uneven participant numbers across the 4 included studies prevented meta-regression and sensitivity analysis, resulting in incomplete uncertainty quantification for this analysis. Fourth, we did not evaluate the cost-effectiveness or real-world implementation of HCVcAg. Alternative laboratory modules, such as point-of-care HCV NAATs, offer superior diagnostic performance, faster turnaround times, and greater utility in treatment response monitoring [51, 52]. Thus, HCVcAg's specific role in HCV elimination strategies and its on-site application in micro-elimination efforts require further investigation as it is not a point-of-care modality and cost-effectiveness analyses are lacking. Nevertheless, our findings provide preliminary evidence supporting potential clinical implementation and establish a foundation for future large-scale studies to verify its practical utility in field settings.

## CONCLUSIONS

Our meta-analysis of 4 studies utilizing the ARCHITECT HCV Ag assay demonstrated a pooled sensitivity of 0.92, specificity of 0.99, PLR of 73.54, and NLR of 0.08 for detecting acute or recent HCV infection among PWH. Given the global and local incidence of HCV infection among PWH, the NPV was as high as 100% in the simulation, indicating its potential for clinical application. These findings suggest that the HCVcAg assay may serve as a valuable tool for integration into clinical practice for HCV screening and diagnostic protocols for this high-risk population, facilitating the implementation of the “treatment-as-prevention” strategies for PWH. Further research is warranted to evaluate the comparative advantage and cost-effectiveness of the HCVcAg assay in this specific context, particularly in comparison with other point-of-care NAAT modules.

## Supplementary Material

ofaf576_Supplementary_Data

## Supplementary Data

Supplementary materials are available at *Open Forum Infectious Diseases* online. Consisting of data provided by the authors to benefit the reader, the posted materials are not copyedited and are the sole responsibility of the authors, so questions or comments should be addressed to the corresponding author.
