# Supplementary material for: HCV Core Antigen Diagnostic Performance for Acute/Recent HCV Infection in People With HIV-1: A Systematic Review and Meta-Analysis
Source: Open Forum Infect Dis. 2025 Oct 17;12(10):ofaf576. doi: 10.1093/ofid/ofaf576 (PMC12531625; doi:10.1093/ofid/ofaf576)
Supplement: ofaf576_Supplementary_Data [file ofaf576_supplementary_data.docx]

**Supplementary Material**

**Figure S1.** Quality assessment of the included studies by the Quality Assessment of Diagnostic Accuracy Studies 2 (QUADAS-2) **
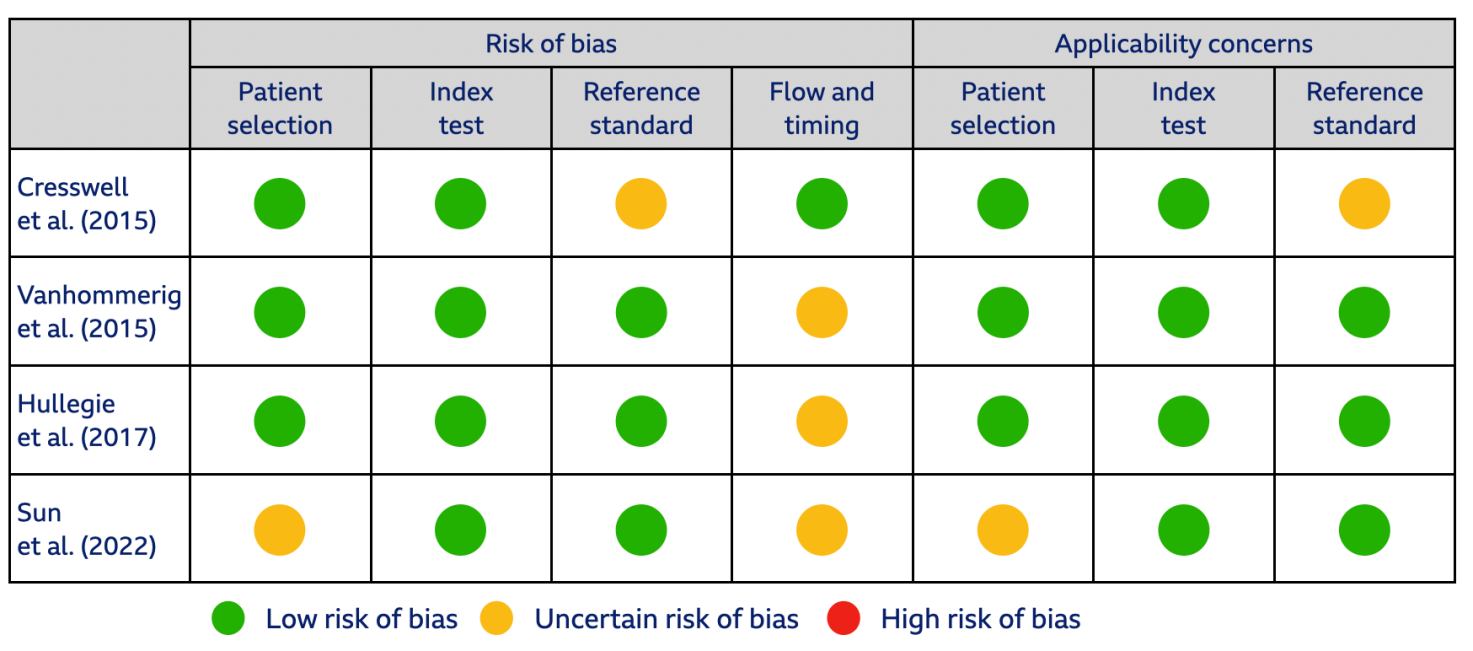
**

**Figure S2.** The results of Fagan plots using (a) the annual global incidence (0.85% [13]) of HCV infection and (b) local (Taiwan, 4.93% [35]) incidence of HCV reinfection among people living with HIV (PLWH) to demonstrate post-test probabilities


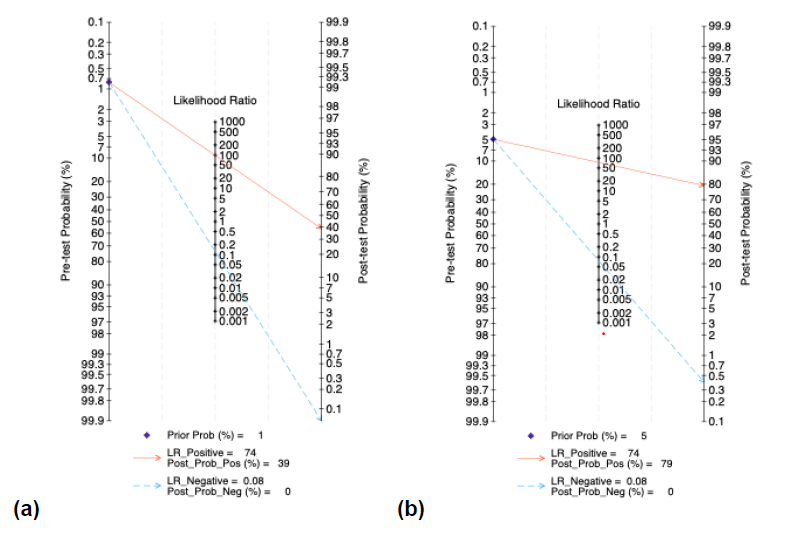


**Table S1.** Inclusion/Exclusion Criteria – Population, Intervention, Comparison, Outcomes and Study Design (PICOS)

| Research Question | | | | |
| --- | --- | --- | --- | --- |
| Is HCV core antigen (HCVcAg) testing able to be used to test acute or recent infection among people living with HIV (PLWH)? | | | | |
| PICO | | Comment | Key words | Searching terms in overview |
| Population | PLWH who used HCVcAg testing to examine acute or recent infection | The definition of acute or recent HCV infection varies among studies. | People with HIV | “HIV” OR “PWH” OR “PLWH” |
| Intervention | HCVcAg test  (Abbott Architect^®^ HCV Ag assay, Abbott, Germany) | Other HCVcAg testing methods were excluded | HCV core antigen | “HCV” AND (“antigen” OR “core antigen” OR “HCV Ag” OR “HCVcAg”) AND “diagnosis” |
| Comparison | Nucleic Acid Amplification Tests as gold standards | Aiming to examine the diagnostic performance of the HCVcAg test | - | - |
| Outcomes | The sensitivity and specificity of the HCVcAg test | Studies failed to provide numbers of true positive, false positive, true negative and false negative were excluded | Diagnosis,  sensitivity, specificity | (“Sensitivity” AND “Specificity”) OR “Diagnosis” OR “Screen” OR “Diagnostic performance” OR “Diagnostic ability” |
| Study design | Case-control, cross-sectional, cohort, or randomized trial studies are included. | Studies not providing timing of HCV infection, study targeting only active HCV infection and descriptive epidemiological studies were excluded | - | - |

**Table S2.** Search Strategy

| Research question | The sensitivity and specificity of HCVcAg test in diagnosis of acute or recent HCV infection among PLWH |
| --- | --- |
| Databases | PubMed, EMBASE, Scopus, Web of Science (WOS) |

| List of sources searched | Search strategy in detail |
| --- | --- |
| PubMed | (("sensitive"[All Fields] OR "sensitively"[All Fields] OR "sensitives"[All Fields] OR "sensitivities"[All Fields] OR "sensitivity and specificity"[MeSH Terms] OR ("sensitivity"[All Fields] AND "specificity"[All Fields]) OR "sensitivity and specificity"[All Fields] OR "sensitivity"[All Fields]) AND ("sensitivity and specificity"[MeSH Terms] OR ("sensitivity"[All Fields] AND "specificity"[All Fields]) OR "sensitivity and specificity"[All Fields] OR "specificity"[All Fields] OR "specific"[All Fields] OR "specifically"[All Fields] OR "specification"[All Fields] OR "specifications"[All Fields] OR "specificities"[All Fields] OR "specifics"[All Fields] OR "specifities"[All Fields] OR "specifity"[All Fields]) AND ("research design"[MeSH Terms] OR ("research"[All Fields] AND "design"[All Fields]) OR "research design"[All Fields] OR "test"[All Fields] OR ("test s"[All Fields] OR "tested"[All Fields] OR "testing"[All Fields] OR "testings"[All Fields] OR "tests"[All Fields]) OR ("diagnosis"[MeSH Subheading] OR "diagnosis"[All Fields] OR "screening"[All Fields] OR "mass screening"[MeSH Terms] OR ("mass"[All Fields] AND "screening"[All Fields]) OR "mass screening"[All Fields] OR "early detection of cancer"[MeSH Terms] OR ("early"[All Fields] AND "detection"[All Fields] AND "cancer"[All Fields]) OR "early detection of cancer"[All Fields] OR "screen"[All Fields] OR "screenings"[All Fields] OR "screened"[All Fields] OR "screens"[All Fields]) OR ("diagnosable"[All Fields] OR "diagnosi"[All Fields] OR "diagnosis"[MeSH Terms] OR "diagnosis"[All Fields] OR "diagnose"[All Fields] OR "diagnosed"[All Fields] OR "diagnoses"[All Fields] OR "diagnosing"[All Fields] OR "diagnosis"[MeSH Subheading]) OR "Diagnostic performance"[All Fields] OR "Diagnostic ability"[All Fields]) AND (("Hepatitis C"[All Fields] OR "HCV"[All Fields]) AND ("acute"[All Fields] OR "acutely"[All Fields] OR "acutes"[All Fields] OR ("recent"[All Fields] OR "recently"[All Fields] OR "recents"[All Fields]))) AND ((("Hepatitis C"[MeSH Terms] OR "Hepatitis C"[All Fields] OR "hepacivirus"[MeSH Terms] OR "hepacivirus"[All Fields] OR "HCV"[All Fields]) AND ("Core"[All Fields] OR ("antigen s"[All Fields] OR "antigene"[All Fields] OR "antigenes"[All Fields] OR "antigenic"[All Fields] OR "antigenically"[All Fields] OR "antigenicities"[All Fields] OR "antigenicity"[All Fields] OR "antigenized"[All Fields] OR "antigens"[MeSH Terms] OR "antigens"[All Fields] OR "antigen"[All Fields]))) OR "HCV Ag"[All Fields] OR "HCVcAg"[All Fields]) AND ("hiv"[MeSH Terms] OR "hiv"[All Fields] OR "PWH"[All Fields] OR "PLWH"[All Fields])) AND (2000:2024[pdat]) |
| EMBASE | ('sensitivity'/exp OR sensitivity) AND ('specificity'/exp OR specificity) AND ('test'/exp OR test OR 'testing'/exp OR testing OR 'screen'/exp OR screen OR 'diagnosis'/exp OR diagnosis OR 'diagnostic performance'/exp OR 'diagnostic performance' OR 'diagnostic ability') AND ('hepatitis c'/exp OR 'hepatitis c' OR 'hcv'/exp OR hcv) AND (acute OR recent) AND (('hepatitis c'/exp OR 'hepatitis c' OR 'hcv'/exp OR hcv) AND ('core'/exp OR core OR 'antigen'/exp OR antigen) OR 'hcv ag' OR hcvcag) AND ('hiv'/exp OR hiv OR pwh OR plwh) AND [2000-2024]/py |
| Scopus | TITLE-ABS-KEY ( ( ( sensitivity ) AND ( specificity ) AND ( ( ( test ) OR ( testing ) ) OR ( screen ) OR ( diagnosis ) OR ( "Diagnostic performance" ) OR ( "Diagnostic ability" ) ) ) AND ( ( ( "Hepatitis C" ) OR ( hcv ) ) AND ( ( acute ) OR ( "recent" ) ) ) AND ( ( ( ( "Hepatitis C" ) OR ( hcv ) ) AND ( ( core ) OR ( antigen ) ) ) OR ( "HCV Ag" ) OR ( hcvcag ) ) AND ( ( hiv ) OR ( pwh ) OR ( plwh ) ) ) AND PUBYEAR > 1999 AND PUBYEAR < 2025 |
| WOS | ((Sensitivity) AND (Specificity) AND (((Test) OR (Testing)) OR (Screen) OR (Diagnosis) OR ("Diagnostic performance") OR ("Diagnostic ability"))) AND ((("Hepatitis C") OR (HCV)) AND ((Acute) OR (Recent))) AND (((("Hepatitis C") OR (HCV)) AND ((Core) OR (Antigen))) OR ("HCV Ag") OR (hcvcah)) AND ((HIV) OR (PWH) OR (PLWH)) (All Fields) |

**Table S3.** Searches in PubMed (Access on 2024/05/14)

| No. (#) | Keywords | Searched results |
| --- | --- | --- |
| Test, screen, diagnosis, diagnostic performance, sensitivity and specificity | | |
| 1 | Sensitivity | 1,789,783 |
| 2 | Specificity | 3,649,385 |
| 3 | Test | 2,841,458 |
| 4 | Testing | 2,546,864 |
| 5 | Screen | 4,469,952 |
| 6 | Diagnosis | 7,774,231 |
| 7 | “Diagnostic performance” | 28,493 |
| 8 | “Diagnostic ability” | 3,258 |
| Acute, recent HCV infection | | |
| 9 | “Hepatitis C” | 86,573 |
| 10 | HCV | 57,991 |
| 11 | Acute | 1,190,736 |
| 12 | Recent | 1,940,396 |
| HCV Core antigen | | |
| 13 | Core | 412,223 |
| 14 | Antigen | 959,428 |
| 15 | “HCV Ag” | 104 |
| 16 | HCVcAg | 68 |
| People living with HIV (PLWH), people with HIV (PWH) | | |
| 17 | HIV | 328,315 |
| 18 | PWH | 2,703 |
| 19 | PLWH | 4,273 |
| Combination of searches | | |
| 20 | #1 AND #2 AND ((#3 OR #4) OR #5 OR #6 OR #7 OR #8) | 726,659 |
| 21 | (#9 OR #10) AND (#11 OR #12) | 15,095 |
| 22 | ((#9 OR #10) AND (#13 OR #14)) OR #15 OR #16 | 12,514 |
| 23 | #17 OR #18 OR #19 | 328,944 |
| Final research results | | |
| 24 | #20 AND #21 AND #22 AND #23 | **28** |

**Table S4.** Searches in Embase (Access on 2024/05/14)

| No. (#) | Keywords | Searched results |
| --- | --- | --- |
| Test, screen, diagnosis, diagnostic performance, sensitivity and specificity | | |
| 1 | Sensitivity | 1,630,976 |
| 2 | Specificity | 949,098 |
| 3 | Test | 3,378,282 |
| 4 | Testing | 1,074,474 |
| 5 | Screen | 240,814 |
| 6 | Diagnosis | 8,194,014 |
| 7 | “Diagnostic performance” | 39,211 |
| 8 | “Diagnostic ability” | 4,557 |
| Acute, recent HCV infection | | |
| 9 | “Hepatitis C” | 164,924 |
| 10 | HCV | 104,612 |
| 11 | Acute | 2,035,702 |
| 12 | Recent | 1,632,518 |
| HCV Core antigen | | |
| 13 | Core | 520,257 |
| 14 | Antigen | 1,285,522 |
| 15 | “HCV Ag” | 233 |
| 16 | HCVcAg | 101 |
| People living with HIV (PLWH), people with HIV (PWH) | | |
| 17 | HIV | 418,512 |
| 18 | PWH | 4,680 |
| 19 | PLWH | 6,310 |
| Combination of searches | | |
| 20 | #1 AND #2 AND ((#3 OR #4) OR #5 OR #6 OR #7 OR #8) | 513,325 |
| 21 | (#9 OR #10) AND (#11 OR #12) | 29,129 |
| 22 | ((#9 OR #10) AND (#13 OR #14)) OR #15 OR #16 | 38,168 |
| 23 | #17 OR #18 OR #19 | 439,643 |
| Final research results | | |
| 24 | #20 AND #21 AND #22 AND #23 | **67** |

**Table S5.** Searches in Scopus (Access on 2024/05/14)

| No. (#) | Keywords | Searched results |
| --- | --- | --- |
| Test, screen, diagnosis, diagnostic performance, sensitivity and specificity | | |
| 1 | Sensitivity | 2,282,759 |
| 2 | Specificity | 1,154,758 |
| 3 | Test | 6,389,235 |
| 4 | Testing | 2,073,908 |
| 5 | Screen | 337,170 |
| 6 | Diagnosis | 2,676,829 |
| 7 | “Diagnostic performance” | 33,010 |
| 8 | “Diagnostic ability” | 4,491 |
| Acute, recent HCV infection | | |
| 9 | “Hepatitis C” | 128,246 |
| 10 | HCV | 65,597 |
| 11 | Acute | 1,569,491 |
| 12 | Recent | 3,136,388 |
| HCV Core antigen | | |
| 13 | Core | 1,291,802 |
| 14 | Antigen | 1,139,229 |
| 15 | “HCV Ag” | 123 |
| 16 | HCVcAg | 66 |
| People living with HIV (PLWH), people with HIV (PWH) | | |
| 17 | HIV | 364,475 |
| 18 | PWH | 2,618 |
| 19 | PLWH | 4,370 |
| Combination of searches | | |
| 20 | #1 AND #2 AND ((#3 OR #4) OR #5 OR #6 OR #7 OR #8) | 432,653 |
| 21 | (#9 OR #10) AND (#11 OR #12) | 20,294 |
| 22 | ((#9 OR #10) AND (#13 OR #14)) OR #15 OR #16 | 22,150 |
| 23 | #17 OR #18 OR #19 | 365,133 |
| Final research results | | |
| 24 | #20 AND #21 AND #22 AND #23 | **30** |

**Table S6.** Searches in Web of Science (Access on 2024/05/14)

| No. (#) | Keywords | Searched results |
| --- | --- | --- |
| Test, screen, diagnosis, diagnostic performance, sensitivity and specificity | | |
| 1 | Sensitivity | 1,419,184 |
| 2 | Specificity | 556,921 |
| 3 | Test | 5,266,189 |
| 4 | Testing | 5,328,422 |
| 5 | Screen | 1,095,550 |
| 6 | Diagnosis | 1,883,513 |
| 7 | “Diagnostic performance” | 32,089 |
| 8 | “Diagnostic ability” | 3,375 |
| Acute, recent HCV infection | | |
| 9 | “Hepatitis C” | 109,439 |
| 10 | HCV | 70,019 |
| 11 | Acute | 1,339,530 |
| 12 | Recent | 2,098,855 |
| HCV Core antigen | | |
| 13 | Core | 1,216,868 |
| 14 | Antigen | 485,717 |
| 15 | “HCV Ag” | 108 |
| 16 | HCVcAg | 56 |
| People living with HIV (PLWH), people with HIV (PWH) | | |
| 17 | HIV | 378,438 |
| 18 | PWH | 3,677 |
| 19 | PLWH | 4,307 |
| Combination of searches | | |
| 20 | #1 AND #2 AND ((#3 OR #4) OR #5 OR #6 OR #7 OR #8) | 187,141 |
| 21 | (#9 OR #10) AND (#11 OR #12) | 13,676 |
| 22 | ((#9 OR #10) AND (#13 OR #14)) OR #15 OR #16 | 12,714 |
| 23 | #17 OR #18 OR #19 | 380,186 |
| Final research results | | |
| 24 | #20 AND #21 AND #22 AND #23 | **12** |

**Table S7. The PRISMA checklist**

| **Section and Topic** | **Item #** | **Checklist item** | **Location where item is reported** |
| --- | --- | --- | --- |
| **TITLE** | | |  |
| Title | 1 | Identify the report as a systematic review. | Page 1, line 1-2 |
| **ABSTRACT** | | |  |
| Abstract | 2 | See the PRISMA 2020 for Abstracts checklist. | Pahe 2-3, line 25-60 |
| **INTRODUCTION** | | |  |
| Rationale | 3 | Describe the rationale for the review in the context of existing knowledge. | Page 5, line 99-101 |
| Objectives | 4 | Provide an explicit statement of the objective(s) or question(s) the review addresses. | Page 5, line 101-104 |
| **METHODS** | | |  |
| Eligibility criteria | 5 | Specify the inclusion and exclusion criteria for the review and how studies were grouped for the syntheses. | Page 7, line 133-142 |
| Information sources | 6 | Specify all databases, registers, websites, organisations, reference lists and other sources searched or consulted to identify studies. Specify the date when each source was last searched or consulted. | Page 6, line 119-127 |
| Search strategy | 7 | Present the full search strategies for all databases, registers and websites, including any filters and limits used. | Supplementary material |
| Selection process | 8 | Specify the methods used to decide whether a study met the inclusion criteria of the review, including how many reviewers screened each record and each report retrieved, whether they worked independently, and if applicable, details of automation tools used in the process. | Page 6, line 128-130 |
| Data collection process | 9 | Specify the methods used to collect data from reports, including how many reviewers collected data from each report, whether they worked independently, any processes for obtaining or confirming data from study investigators, and if applicable, details of automation tools used in the process. | Page 8, line 159-166 |
| Data items | 10a | List and define all outcomes for which data were sought. Specify whether all results that were compatible with each outcome domain in each study were sought (e.g. for all measures, time points, analyses), and if not, the methods used to decide which results to collect. | Page 8, line 159-166 |
|  | 10b | List and define all other variables for which data were sought (e.g. participant and intervention characteristics, funding sources). Describe any assumptions made about any missing or unclear information. | Page 7, line 148-151; page 8, line 159-166 |
| Study risk of bias assessment | 11 | Specify the methods used to assess risk of bias in the included studies, including details of the tool(s) used, how many reviewers assessed each study and whether they worked independently, and if applicable, details of automation tools used in the process. | Page 8, line 159-166 |
| Effect measures | 12 | Specify for each outcome the effect measure(s) (e.g. risk ratio, mean difference) used in the synthesis or presentation of results. | Page 8, line 159-161 |
| Synthesis methods | 13a | Describe the processes used to decide which studies were eligible for each synthesis (e.g. tabulating the study intervention characteristics and comparing against the planned groups for each synthesis (item #5)). | Page 8, line 159-166 |
|  | 13b | Describe any methods required to prepare the data for presentation or synthesis, such as handling of missing summary statistics, or data conversions. | Page 8, line 159-161 |
|  | 13c | Describe any methods used to tabulate or visually display results of individual studies and syntheses. | Page 8, line 159-161 |
|  | 13d | Describe any methods used to synthesize results and provide a rationale for the choice(s). If meta-analysis was performed, describe the model(s), method(s) to identify the presence and extent of statistical heterogeneity, and software package(s) used. | Page 8, line 159-161  Page 8, line 166-169 |
|  | 13e | Describe any methods used to explore possible causes of heterogeneity among study results (e.g. subgroup analysis, meta-regression). | Page 8, line 167-169 |
|  | 13f | Describe any sensitivity analyses conducted to assess robustness of the synthesized results. | Page 14, line 322-323 |
| Reporting bias assessment | 14 | Describe any methods used to assess risk of bias due to missing results in a synthesis (arising from reporting biases). | Not applicable |
| Certainty assessment | 15 | Describe any methods used to assess certainty (or confidence) in the body of evidence for an outcome. | Not applicable |
| **RESULTS** | | |  |
| Study selection | 16a | Describe the results of the search and selection process, from the number of records identified in the search to the number of studies included in the review, ideally using a flow diagram. | Figure 1 |
|  | 16b | Cite studies that might appear to meet the inclusion criteria, but which were excluded, and explain why they were excluded. | Figure 1 |
| Study characteristics | 17 | Cite each included study and present its characteristics. | Page 10, line 194-211 |
| Risk of bias in studies | 18 | Present assessments of risk of bias for each included study. | Figure S1 |
| Results of individual studies | 19 | For all outcomes, present, for each study: (a) summary statistics for each group (where appropriate) and (b) an effect estimate and its precision (e.g. confidence/credible interval), ideally using structured tables or plots. | Table 1 |
| Results of syntheses | 20a | For each synthesis, briefly summarise the characteristics and risk of bias among contributing studies. | Page 10, line 194-211 |
|  | 20b | Present results of all statistical syntheses conducted. If meta-analysis was done, present for each the summary estimate and its precision (e.g. confidence/credible interval) and measures of statistical heterogeneity. If comparing groups, describe the direction of the effect. | Page 11, line 224-240 |
|  | 20c | Present results of all investigations of possible causes of heterogeneity among study results. | Page 11, line 242-247 |
|  | 20d | Present results of all sensitivity analyses conducted to assess the robustness of the synthesized results. | Page 14, line 322-323 |
| Reporting biases | 21 | Present assessments of risk of bias due to missing results (arising from reporting biases) for each synthesis assessed. | Not applicable |
| Certainty of evidence | 22 | Present assessments of certainty (or confidence) in the body of evidence for each outcome assessed. | Not applicable |
| **DISCUSSION** | | |  |
| Discussion | 23a | Provide a general interpretation of the results in the context of other evidence. | Page 11, line 237-241 |
|  | 23b | Discuss any limitations of the evidence included in the review. | Page 14, line 314-336 |
|  | 23c | Discuss any limitations of the review processes used. | Page 14, line 314-336 |
|  | 23d | Discuss implications of the results for practice, policy, and future research. | Page 14, line 314-336 |
| **OTHER INFORMATION** | | |  |
| Registration and protocol | 24a | Provide registration information for the review, including register name and registration number, or state that the review was not registered. | Page 16, line 340-346 |
|  | 24b | Indicate where the review protocol can be accessed, or state that a protocol was not prepared. | Not applicable |
|  | 24c | Describe and explain any amendments to information provided at registration or in the protocol. | Not applicable |
| Support | 25 | Describe sources of financial or non-financial support for the review, and the role of the funders or sponsors in the review. | Page 17, line 357-361 |
| Competing interests | 26 | Declare any competing interests of review authors. | Page 17, line 357-361 |
| Availability of data, code and other materials | 27 | Report which of the following are publicly available and where they can be found: template data collection forms; data extracted from included studies; data used for all analyses; analytic code; any other materials used in the review. | Supplementary material |

*From:*  Page MJ, McKenzie JE, Bossuyt PM, Boutron I, Hoffmann TC, Mulrow CD, et al. The PRISMA 2020 statement: an updated guideline for reporting systematic reviews. BMJ 2021;372:n71. doi: 10.1136/bmj.n71. This work is licensed under CC BY 4.0. To view a copy of this license, visit <https://creativecommons.org/licenses/by/4.0/>
